# Legalizing Marijuana in Canada — A Double-Edged Sword: A Response to Recent Commentaries

**DOI:** 10.15171/ijhpm.2016.152

**Published:** 2016-12-14

**Authors:** Mohammad Hajizadeh

**Affiliations:** School of Health Administration, Dalhousie University, Halifax, NS, Canada.

I am very grateful to Rehm and colleagues^1^ and Lake and Kerr^2^ for their interesting and insightful commentaries on my editorial *“Legalizing and regulating marijuana in Canada: review of potential economic, social and health impacts.”*^3^ In their responses, Lake and Kerr^2^ offer additional insights and considerations on the public health implications of recreational marijuana legalization and Rehm and colleagues^1^ explain that economic, social, and health impacts of the legalization will largely depend on the design and implementation of appropriate legal and regulatory framework.

Lake and Kerr^2^ highlight the underdeveloped and equivocal body of scientific literature on the two most ‘commonly’ cited public health concerns of legalizing marijuana, viz., adolescent usage and impaired driving. They also discuss some possible public health benefits of legalizing marijuana. Specifically, they emphasize the possible health benefits associated with substituting marijuana for drugs that cause more harm. Rehm and colleagues,^1^ on the other hand, focus on the main behavioural drivers of marijuana-related health harms to elaborate how the details of regulations for recreational marijuana contribute to a reduction in risky health behaviours. Additionally, they provide some further insights on the issues related to the production and sale of legal marijuana and complications related to the existence of medical marijuana in Canada.

I agree with Lake and Kerr^2^ view on the fact that the current studies on the health impacts of marijuana (eg, issues of adolescent usage and impaired driving) limit our ability to predict the extent of health consequences of legalization in Canada. Nonetheless, I believe that, in the absence of appropriate regulations, legalization of marijuana can potentially contribute to marijuana-impaired driving problem. Although there is a high level of heterogeneity regarding quality and findings of studies measuring the association between marijuana use and motor vehicle collision, the most recent systematic reviews and meta-analysis of all related studies indicate an effect of acute marijuana intoxication on motor vehicle crash risk.^4,5^ I also agree with Lake and Kerr^2^ point that the issues of adolescent usage in Canada may not be a big health concern since the current literature indicates that marijuana is easily accessible among youth in Canada and the current literature does not provide clear evidence on the effect of legalization on the adolescent usage. Nevertheless, the legalization can potentially increase accessibility of this drug among adolescents if the government does not design and implement regulations to keep the drug out of the hands of minors. The legalization may also lead to an increase in the overall consumption of the drug because it will substantially reduce the price of marijuana and make it easier and safer to obtain the drug, which is legal and more socially acceptable.^6^ Reduction in the price of marijuana, like other substances such as alcohol and tobacco,^7-9^ may lead to an increase in marijuana use.^10^ An increase in the overall consumption of marijuana, in turn, can potentially lead to an increase in the health burden of marijuana use. However, it should be noted that it is difficult to predict how much the price of legal marijuana will drop, or how much consumers may increase their marijuana use in response to the price change in the market.^11,12^

I fully agree with Rehm and colleagues^1^ when they write “the devil will be in the details” and “the true challenge will be the exact implementation.” I believe that legalizing marijuana is a double-edged sword with positive and negative consequences. If the law and regulations of recreational marijuana are carefully introduced by the federal government and implemented at the provincial and municipal levels based on available scientific evidence, Canada can manage public health issues associated with legal marijuana while generating economic benefits through savings in law enforcement costs and increased tax revenue from legal drugs and social benefits by reducing the size of Canada’s black market and its consequences for the society. As Lake and Kerr^2^ emphasize in their commentary, “we must ensure the policy is based on the science that has been presented fairly within the context of all evidence.”

## Ethical issues

Not applicable.

## Competing interests

Author declares that he has no competing interests.

## Author’s contribution

MH is the single author of the paper.
